# 

**Published:** 1887-07

**Authors:** 


Vol. VIII.] PUBLISHED QUARTERLY AT TWO DOLLARS A YEAR. SINGLE COPIES, 60 CENTS. [No. 3.

### THE JOURNAL OF COMPARATIVE MEDICINE AND SURGERY.

LIBRARY INDEXED S.G.O.

EDITED BY
W. A. CONKLIN, Ph. D., D. V. S.,
DIRECTOR OF ZOOLOGICAL GARDENS, NEW YORK CITY.
RUSH SHIPPEN HUIDEKOPER, M. D., Veterinarian (*Alfort*),
DEAN OF VETERINARY DEPARTMENT, UNIVERSITY OF PENNSYLVANIA.

COLLABORATORS.

COMPARATIVE ANATOMY AND PHYSIOLOGY.
PROF. JOSEPH LEIDY, M.D., L.L.D., . University of Penna.
PROF. HENRY C. CHAPMAN, M.D., . Jefferson Med. College.
PROF. E. C. SPITZKA, M.D., . . . . . . . . New York City.
PROF. R. MEADE SMITH, M.D., . . . University of Penna.
PROF. J. T. DUNCAN, M.D., V.S., . Ontario Veterinary College.
PROF. C. M. O'LEARY, M.D., L.L.D., . . . New York City.

COMPARATIVE PATHOLOGY.
PROF. ALBERT JOHNE, . . Royal Vet. School, Dresden, Ger.
PROF. JAMES TYSON, M.D., . . . . University of Penna.
PROF. R. H. FITZ, M.D., . . . . . . Harvard University.
PROF. WM. OSLER, M.D., . . . . . University of Penna.
E. O. SHAKESPEARE, M.D., . . . . Blockley Hospital, Phila.
A. W. CLEMENT, V. S., . . . . . . . . Montreal Vet. College.
THOMAS B. ROGERS, D. V. S., . . . . University of Penna.

COMPARATIVE SURGERY.
PROF. JAMES LAW, F.R.C.V.S., . . . . Cornell University.
PROF. C. P. LYMAN, F.R.C.V.S., . . . Harvard University.
PROF. D. McEACHRAN, F.R.C.V.S., . . Montreal Vet. College.
PROF. A. T. YOKURA, V.S., . . Imperial School, Tokio, Japan.
PROF. JAMES L. ROBERTSON, M.D., V.S., Am. Vet. College.
PROF. WILLIAM L. ZUILL, M.D., D.V.S., University of Penna.

A. L. HUMMEL, M. D., PUBLISHER,
8 SPRUCE STREET, NEW YORK, and
No. 1217 Filbert Street, Philadelphia, Pa.
LONDON: BALLIERE, TINDALL & COX.
Entered at the Post-Office, New York, N. Y., as second-class mail matter.

